# Potential Beneficial Effects and Pharmacological Properties of Ergosterol, a Common Bioactive Compound in Edible Mushrooms

**DOI:** 10.3390/foods12132529

**Published:** 2023-06-29

**Authors:** Panthakarn Rangsinth, Rajasekharan Sharika, Nattaporn Pattarachotanant, Chatrawee Duangjan, Chamaiphron Wongwan, Chanin Sillapachaiyaporn, Sunita Nilkhet, Nichaporn Wongsirojkul, Anchalee Prasansuklab, Tewin Tencomnao, George Pak-Heng Leung, Siriporn Chuchawankul

**Affiliations:** 1 Department of Transfusion Medicine and Clinical Microbiology, Faculty of Allied Health Sciences, Chulalongkorn University, Bangkok 10330, Thailand; ptkrs@hku.hk (P.R.); sharikarpillai@gmail.com (R.S.); nichaporn.w@chula.ac.th (N.W.); 2 Department of Pharmacology and Pharmacy, The University of Hong Kong, Pokfulam, Hong Kong SAR, China; 3 Department of Clinical Chemistry, Faculty of Allied Health Sciences, Chulalongkorn University, Bangkok 10330, Thailand; nat.ahs11@gmail.com (N.P.); chamaiphorn.wongwan@gmail.com (C.W.); chanin.s@chula.ac.th (C.S.); 6273006537@student.chula.ac.th (S.N.); tewin.t@chula.ac.th (T.T.); 4 Leonard Davis School of Gerontology, University of Southern California, Los Angeles, CA 90089, USA; duangjan@usc.edu; 5 College of Public Health Sciences, Chulalongkorn University, Bangkok 10330, Thailand; anchalee.pr@chula.ac.th; 6 Immunomodulation of Natural Products Research Unit, Chulalongkorn University, Bangkok 10330, Thailand

**Keywords:** ergosterol, nutrition, food chemistry, bioactive compound, nutraceutical

## Abstract

Ergosterol is an important sterol commonly found in edible mushrooms, and it has important nutritional value and pharmacological activity. Ergosterol is a provitamin. It has been well established that edible mushrooms are an excellent food source of vitamin D2 because ergosterol is a precursor that is converted to vitamin D2 under ultraviolet radiation. The pharmacological effects of ergosterol, which include antimicrobial, antioxidant, antimicrobial, anticancer, antidiabetic, anti-neurodegenerative, and other activities, have also been reported. This review aims to provide an overview of the available evidence regarding the pharmacological effects of ergosterol and its underlying mechanisms of action. Their potential benefits and applications are also discussed.

## 1. Introduction

Natural bioactive compounds are widely used as complementary medicines, including in dietary supplements. Their potential applications have been investigated for several decades. Researchers have identified novel biomolecules with desirable pharmacological properties from natural products, optimized their efficacy and safety using medicinal chemistry and pharmaceutical science, and developed them into pharmaceutical or nutraceutical products for the treatment or prevention of several diseases. Most natural compounds have been isolated from edible sources or used in traditional medicines worldwide for many years; therefore, they are supposed to be relatively safe.

Edible mushrooms are rich in proteins, dietary fiber, vitamins, minerals, and many bioactive substances such as polysaccharides, terpenoids, polyphenols, alkaloids, lactones, and sterols [1,2]. The major type of sterol in edible mushrooms is ergosterol [3]. Ergosterol has become an increasingly popular topic in pharmacological research because of its potential health benefits and widespread presence in various foods and dietary products. Its pharmacological effects on oxidation, immune function, diabetes, cancer, and other diseases have been previously reported. However, there is hitherto no review summarizing these effects and discussing their potential implications.

This review is aimed at outlining an overview of the available evidence proving the pharmacological effects of ergosterol and its underlying mechanisms of action. Its potential benefits and therapeutic applications are also discussed. The information inside this review is based on the scientific literature searched and retrieved from the PubMed and Google Scholar databases.

## 2. Overview of Ergosterol Structure

**Figure 1:**
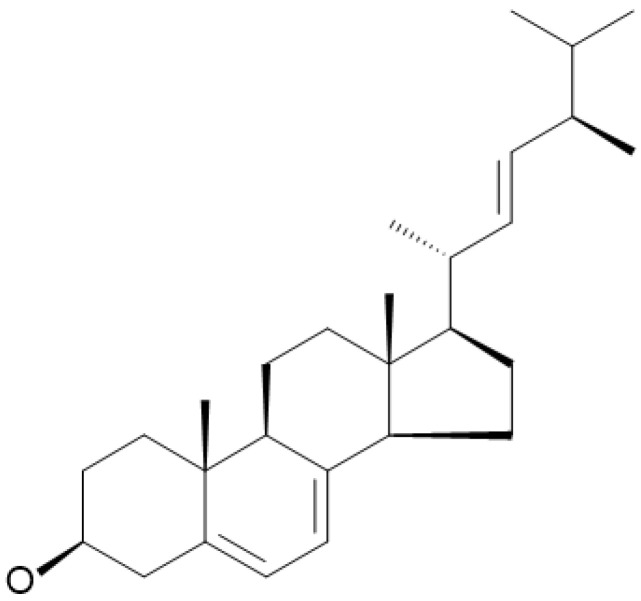
Structure of ergosterol.

Ergosterol, or ergosta-5,7,22-trien-3β-ol, is the most abundant sterol in the cell membrane of fungi, including edible mushrooms. It maintains fungal cell membrane integrity, similar to cholesterol in animal cell membranes [4]. This compound contains three double bonds and β-hydroxy groups at positions 5, 7, and 22 with a 1,2-cyclopentanoperhydrophenanthrene ring nucleus (Figure 1), making it behave like an amphipathic lipid [5]. Ergosterol exists in free and esterified forms. When exposed to ultraviolet light (280–320 nm), it undergoes photolysis. During photolysis, it is transformed into pre-vitamin D2 (pre-ergocalciferol), followed by thermal conversion into vitamin D2 (ergocalciferol), which is essential for human nutrition. After consumption, vitamin D2 is converted to 25-hydroxyvitamin D through hepatic metabolism [6]. It is then transported to the kidney, where it is further transformed into 1,25-dihydroxyvitamin D, which is known as calcitriol, the active form in living organisms that plays important roles in calcium homeostasis and bone health [7].

## 3. Natural Sources of Ergosterol

Ergosterol is found in various sources but is abundant in fungi, particularly mushrooms [8]. Ergosterol content varies among different species and parts of mushrooms [9,10]. The contents of ergosterol are 3.52 mg/g and 0.43 mg/g in button mushrooms and morel mushrooms, respectively [11]. The contents of ergosterol in fresh white and brown button mushrooms are 0.446 mg/g and 0.394 mg/g, respectively [12], whereas the contents of ergosterol in the caps of dry white and brown button mushroom are 3.30–3.76 mg/g and 2.71–4.56 mg/g, respectively [9]. Ergosterol content is reduced with aging of the mushrooms. The mushroom cap contains higher ergosterol content than the stem did at the same growth stage [9]. Although ergosterol is found in many edible mushrooms, it has been reported to be present in some plant extracts including Chinese hickory, hazelnut, lupin, cactus, and firethorn [13,14,15,16]. However, it is unclear whether the ergosterol is a real metabolite in these plants or if its presence in plant extracts is due to the contamination of fungi so further study is required. The dietary sources of ergosterol from mushrooms are summarized in Table 1.

## 4. Pharmacokinetics, Drug-Likeness, and Toxicity of Ergosterol

The pharmacokinetic characteristics of ergosterol were determined using a rat model [38]. In the rats that were orally administered ergosterol for 36 h, the area under the plasma drug concentration time curve at 0–36 h was 22.29 ± 5.08 µg‧h/mL, the half-life was 5.90 ± 1.41 h, the maximum observed concentration (C_max_) was 2.27 ± 0.19 µg/mL, and the time to C_max_ was 8.00 ± 1.18 h. Approximately 62.5% of the administered ergosterol was excreted in feces, whereas 3.2% of the ergosterol was eliminated in urine.

The oral bioavailability of ergosterol is not known because the intravenous injection of ergosterol has not been carried out in a pharmacokinetic study for the calculation of bioavailability. However, the oral bioavailability is expected to be low, as reflected from the long amount of time required for achieving C_max_ and because of the poor aqueous solubility of ergosterol. However, the bioavailability and effectiveness of ergosterol can be further enhanced using a special drug delivery system. Interestingly, one study demonstrated that the use of nanostructured lipid carriers (NLCs) can improve the solubility and bioavailability of ergosterol. A pharmacodynamic study has also confirmed that the inhibitory effects of ergosterol-loaded NLCs on high-glucose-stimulated mesangial cell proliferation and extracellular matrix (ECM) accumulation were stronger than those of raw ergosterol [39]. Similarly, the oral bioavailability of ergosterol can be increased after encapsulation in PGLA nanoparticles [40].

The drug-likeness and toxicity of several bioactive compound from mushrooms, including ergosterol, were previously assessed using an in silico approach by Rangsinth et al. [41]. According to Lipinski’s rule, ergosterol exhibited drug-like properties, including a molecular weight ≤ 500, number of hydrogen bond acceptors ≤ 10, number of hydrogen bond donors ≤ 5, and Log P_o/w_ ≤ 5. Additionally, ergosterol did not cause hepatotoxicity or skin allergy, and its predicted median lethal dose in rats was 2.05 mol/kg [41]. Moreover, without the functional groups which confer as carcinogenic or mutagenic as indicated by the Benigni–Bossa rule, ergosterol was assumed to be non-carcinogenic and non-mutagenic.

## 5. Antioxidant Activity

The imbalance between the generation and removal of free radicals leads to increased levels of reactive oxygen species (ROS) and reactive nitrogen species (RNS), which play crucial roles in oxidative stress, and hence, the aging process [42]. ROS and RNS such as superoxide (O2^•−^), hydroxyl (OH^•^), nitric oxide (NO^•^), and nitrogen dioxide (NO2^•^) radicals are byproducts of biological metabolism [43]. Excessive oxidative stress results in damage to macromolecules, including DNA, cell membranes, and proteins, leading to the development of various diseases such as cancer, atherosclerosis, kidney disease, diabetes, and neurodegenerative diseases [42,43].

The outcome and mechanism of the antioxidant effect of ergosterol are summarized in Table 2. For instance, ergosterol exhibits antioxidant properties that contribute to the resistance of yeast to tert-butyl hydroperoxide-induced free radicals [44]. Ergosterol reacts with hydrogen peroxide (H_2_O_2_), thereby inhibiting lipid peroxidation and reducing the intracellular ROS level [45]. Ergosterol treatment can markedly reduce rat myocardial injury induced by lipopolysaccharide (LPS) injection via its antioxidant and anti-apoptotic effects, which are modulated through an Nrf2 signaling-dependent mechanism [46]. Recently, ergosterol-rich *Auricularia polytricha* mushroom extract showed antioxidant defense against oxidative-stress-induced neurotoxicity by up-regulating mRNA expression of several antioxidant enzymes [20].

## 6. Anti-Inflammatory Activity

Inflammation is a biological reaction that occurs when body parts are exposed to harmful or irritating stimuli. Various cytokines are generated at inflammatory sites as mediators that remove damaged cells, pathogens, or harmful stimuli. Therefore, the initiation of the healing process is important. However, exaggerated and uncontrolled inflammation is a key characteristic and risk factor related to the pathogenesis of many diseases, including atherosclerosis, obesity, metabolic syndrome, diabetes, cancer, and neurodegenerative disorders [47].

Ergosterol-containing mushrooms suppress the expression of inflammatory mediators through several signaling pathways. One such pathway is the Janus kinase-signal transducer and activator of transcription (JAK-STAT) pathway, which is involved in cell division, cell death, tumor formation, immunomodulation, and inflammation. Ergosterol interferes with the expression of JAK3/STAT3 genes in cigarette-smoke-induced chronic obstructive pulmonary disease (COPD) in ICR mice. As a result, ergosterol effectively ameliorates COPD severity by inhibiting the pro-inflammatory cytokines [48]. Similar results were observed in the same COPD models using 16HBE cells and Balb/c mice [49]. Ergosterol peroxide, purified from the edible mushroom *Sarcodon aspratus*, exerts anti-inflammatory responses in RAW264.7 macrophages by downregulating the expression of low-density lipoprotein receptors and suppressing STAT1-mediated inflammatory responses in HT29 cells [50]. In addition, ergosterol and ergosterol peroxide inhibit LPS-induced inflammation, along with the phosphorylation of p38, c-Jun amino-terminal kinase (JNK), extracellular signal-regulated protein kinase (ERK), and mitogen-activated protein kinases (MAPKs) [51]. Ergosterol acetate also suppresses the phosphorylation of ERK in LPS-induced inflammation in RAW246.7 cells [52]. Molecular docking studies have shown that both ergosterol and ergosterol peroxide interact with the ATP-binding site of p38, resulting in the blockade of p38 MAPK phosphorylation, but have no effect on ERK and JNK [53].

Moreover, ergosterol and its derivatives inhibit 5-lipooxygenase (5-LOX) activity, thereby reducing the inflammatory response [54,55]. Ergosterol also suppresses inducible nitric oxide synthase (iNOS) and cyclooxygenase (COX)-2 expression in LPS-induced RAW 264.7 macrophages [52,56]. Consistently, the ergosterol-enriched sub-fraction of an entomopathogenic fungus *Cordyceps militaris* exerts neuroprotective effects by attenuating LPS-activated NO production in BV2 microglial cells [57]. Ergosterol also alleviates the symptoms of LPS-induced acute lung injury in mice by downregulating COX-2 expression [58]. Another study also reported that ergosterol suppresses mRNA expression of COX-2 in rat bladders [59].

Ergosterol and its metabolites, ergosterol peroxide and dihydroergosterol, suppress LPS-induced TNF-α release and interleukin (IL)-1α/β expression in RAW 264.7 macrophage cell lines [27,50,60]. Ergosterol also downregulates TNF-α expression in LC3-associated phagocytosis-induced RAW 264.7 macrophages [61]. Similarly, a dairy product fermented with *Penicillium candidum* which contained ergosterol and dihydroergosterol exhibited an anti-neuroinflammatory effect on LPS-treated microglial cells, that was induced by reducing the expression of TNF-α and interferon (IFN)-γ, suggesting its potential in delaying the onset of dementia [62]. Moreover, ergosterol ameliorates renal inflammation in diabetic mice with streptozotocin-induced nephropathy by reducing inflammatory cytokine levels (e.g., IL-6, TNF-α, and monocyte chemotactic protein factor [MCP]-1), fasting blood glucose level, and renal injury [63].

Ergosterol exerts its anti-neuroinflammatory activity via the TLR4/NF-κB-dependent pathway. Therefore, exploring the potential for developing ergosterol into a novel drug for the treatment of Alzheimer’s disease is viable [64]. It has been reported that the anti-inflammatory functions of ergosterol are mediated by the suppression of transcriptional activity in propidium monoazide-treated human chondrosarcoma (SW1353) cells [65]. Ergosterol and ergosterol peroxide also inhibit NF-κB luciferase activity in RAW246.7 macrophages [27]. Moreover, both ergosterol and ergosterol peroxide bind directly to the active site of NF-κB p65 to restrain the phosphorylation and degradation of IκB-α and thus block the phosphorylation of NF-κB p65 [53]. Furthermore, ergosterol peroxide displays a significant anti-inflammatory effect on LPS-induced human monocytic cells through the inhibition of MyD88 (which is a central node of the inflammatory signaling pathway), VCAM-1 expression, and cytokine (IL-1β, IL-6, and TNF-α) production. This compound also inhibits NF-κB p65 activation effectively [66].

Ergosterol attenuates the symptoms of dextran sulfate sodium-induced colitis in mice, as indicated by the disease activity index, which is reflected in weight loss, severity of diarrhea, and shortening of the colon [67]. The effect of ergosterol on dextran sulfate sodium-induced colitis was also mediated through the suppression of the NF-κB signaling pathway [67]. Furthermore, it was recently found that *Hericium erinaceus*, a medicinal mushroom which contains ergosterol, can cause a significant decrease in the MDA, NO, MPO, NF-κB, IL-6, and TNF-α levels in trinitrobenzene sulfonic acid-induced colitis in mice [68]. Additionally, the pathogenesis of ulcerative colitis is related to mucosal inflammation due to the accumulation of mast cells in the colonic mucosa. Under physiological conditions, the surface of mast cells expresses high-affinity immunoglobulin E (IgE) receptors (FcεRI). The binding of IgE to FcεRI can trigger mast cells to release many inflammatory mediators, including β-hexosaminidase and histamines, which destroy the colonic mucosa. Ergosterol inhibits the aggregation of FcεRI, which is the first step in mast cell activation, and reduces IL-4 and TNF-α mRNA expression in IgE-sensitized RBL-2H3 basophilic leukemia cells [24,25]. Moreover, ergosterol significantly inhibits the activities of β-hexosaminidase and mucosal-type murine bone-marrow-derived mast cells [69]. Similarly, both the ergosterol-rich extract of *Grifola frondosa* and pure ergosterol inhibit histamine release in air pouch-type allergic inflammation and reduce vascular permeability and edema in mice [24,25]. The extract of edible mushroom *Grifola frondosa* and its ergosterol also prevent pollen-allergy-induced ocular itching in mice by suppressing leukotriene B_4_ production in mast cells [70].

## 7. Anticancer Activity

Although the overall survival rate of cancer patients has increased, cancer remains one of the most common causes of morbidity, mortality, and economic burden. Advanced stage cancer and lethal malignancies may not be completely eradicated by current clinical interventions, such as surgery, radiotherapy, chemotherapy, and targeted therapy. The potent anticancer activities of some natural compounds from terrestrial and marine sources have been identified. Paclitaxel, which is isolated from the Pacific yew, is one of the best examples of a natural compound that has been successfully used in clinical practice [71]. Other examples include vinca alkaloids, such as vinblastine, vincristine, and podophyllotoxin [72]. The chemopreventive and chemotherapeutic properties of natural compounds have led to a new era in anticancer drug research. The isolation and characterization of these natural compounds may be more economical than the development of synthetic compounds [73].

Ergosterol has also been reported to exhibit potent anticancer activity (Table 3). One study showed that some lipid-enriched fractions of the mushroom *Ganoderma lucidum*, which are rich in ergosterol derivatives, exhibited cytotoxicity in MDA-MD-231 and HepG2 cell lines but had no effect on normal cells, reflecting the anticancer potential of ergosterol derivatives [74]. Consistently, ergosterol, 5,6-dehydroergosterol, and ergosterol peroxide also exhibit anticancer activity in triple-negative breast cancer cell lines. In addition, ergosterol peroxide induces caspase 3/7-mediated apoptosis, cell cycle arrest, and PARP cleavage. It also inhibits the expression of ATK1, ATK2, Bcl-xL, cyclin D1, and c-Myc, thereby attenuating cancer invasiveness [23]. Ergosterol downregulates signaling proteins such as EGFR, MEK5, AKT1, Smad3, TAB1, NF-κβ, and HIF-α but upregulates p-p38α, pERK-1/2, JNK, fibronectin, p27, and pJNK [75]. Furthermore, ergosterol peroxide induces autophagy, triggers ROS-mediated apoptosis, and attenuates the proliferation and migration of lung cancer A549 cells [75].

In vitro studies on LNCap and DU-145 prostate cancer cell lines further confirmed the androgen-receptor-mediated action of ergosterol. In addition, ergosterol showed anti-proliferative activity against MCF7, NCI-H460, and HeLa cells [76]. Ergosterol inhibits the estrogen receptor in MCF7 breast cancer cell lines [77], probably by inducing apoptosis and S-phase cell cycle arrest [78]. Ergosterol also inhibits Ras expression, the ERK-NADPH oxidase-dependent pathway, and ROS-mediated chromosomal and oxidative damage caused by carcinogen-induced breast cancer [79]. Interestingly, ergosterol may produce synergistic anticancer effects with other drugs such as amphotericin B [80,81]. Ergosterol enhances the anticancer effects of cisplatin. Liposomes modified with arginine-glycine-aspartic acid and octa arginine peptide-mediated drug delivery of ergosterol and cisplatin exhibited strong inhibitory activity against A549 cells [75].

The anticancer effects of ergosterol have also been demonstrated in vivo. Ethanol and chloroform extracts of mushroom *Amauroderma rude*, which contain ergosterol as the main bioactive component, suppress tumor growth in Balb/c mice inoculated with B16 melanoma cancer cells. These effects may be mediated through the activation of FOXO-3, which induces the upregulation of various tumor suppressors, including pro-apoptotic genes, such as FasL, Bad, Bim, and Trail [82]. The oral administration of ergosterol efficiently inhibits bladder cancer in rats by suppressing cyclin D1 expression and, subsequently, COX2 expression; COX2 is a key player involved in the development of bladder cancer [59]. Ergosterol can be converted to brassicasterol, a metabolite that can be retained in the circulatory system for a longer period. The suppression of bladder carcinogenesis may be attributed to the antagonistic effect of brassicasterol on androgen receptors, owing to its structural similarity to testosterone [83]. In silico analysis revealed that ergosterol shows an interaction similar to that of 5α-dihydrotestosterone and testosterone in the binding mode with androgen receptors during a molecular dynamic simulation [84]. Therefore, ergosterol is particularly effective against sex-hormone-dependent cancers.

In a pioneering study, oral administration of ergosterol inhibited Matrigel-induced neovascularization in female C57BL/6 mice, suggesting that it may be an antiangiogenic compound [85]. Ergosterol also downregulates positive modulators of angiogenesis such as VEGFC and STAT3 [86]. These antiangiogenic effects may also contribute to the anticancer effects of ergosterol.

**Table 3:** Anticancer effects of ergosterol.

Cancer	Model	Dose	Activity and Mechanism	Reference
Bladder	*N*-butyl-*N*-(4-hydroxybutyl)nitrosamine-induced bladder cancer in Wistar rats	15 μg/kg/day for 3 weeks	Modulate inflammation-related signaling and inhibit androgen signaling pathways	[59]
		A diet which contains ergosterol 0.01–0.1% for 25 weeks	Inhibit androgen signaling	[83]
Breast	Carcinogen-induced normal breast cell lines MCF10A and MCF12A	1–50 µM	Block carcinogen-induced ROS, ERK activation, DNA oxidation, and DNA damage.	[79]
	MCF7 breast cancer cell lines	IC_50_ = 112.65 μM	Induce S-phase cell cycle arrest and apoptosis	[77,78]
Liver	Hep3B and HepJ5 human hepatocellular carcinoma cells	IC_50_ of Hep3B and HepJ5 cells from 14.54–6.66 μM and 18.65–4.07 μM, respectively, when combined with amphotericin B (5–25 μM)	Increase ROS and LC3-II levels	[81]
Prostrate	LNCaP human prostate adenocarcinoma cell	IC_50_ = 14.68 ± 1.01 μM	Inhibit androgen receptor	[84]
Sarcoma	Sarcoma 180-bearing mice	400 and 800 mg/kg for 20 days	N/A	[85]
Tumor	Matrigel-induced neovascularization in C57BL/6 mice	5, 10 and 20 mg/kg for 5 days	Inhibit angiogenesis	[85]

## 8. Antidiabetic Effects

Diabetes mellitus is a chronic health condition in which blood glucose levels are persistently elevated due to insufficient secretion of insulin from the pancreas or a decreased tissue response to insulin [87]. Nephropathy is the most common and severe complication of diabetes mellitus. It is characterized by irreversible damage to kidney function resulting from hyperglycemia-induced oxidative stress and ECM deposition, which causes renal interstitial fibrosis [88].

Ergosterol derived from the edible mushroom *Pleurotus ostreatus* exhibited promising antidiabetic activity [89]. Ergosterol treatment was able to lower blood glucose in type 2 diabetes mellitus mice and stimulated GLUT4 translocation via the PI3K/Akt pathway and PKC pathway [89]. Ergosterol alleviates diabetic nephropathy in streptozotocin-injected mice with significant correction of biochemical parameters such as plasma levels of glucose, uric acid, creatinine, triglyceride, and total cholesterol. Furthermore, the levels of proteins related to PI_3_K/Akt/NF-κB p65 signaling and cytokines (including IL-6, TNF-α, and MCP-1) [63], as well as to renal pathological changes, were restored by ergosterol treatment [90]. PI_3_K/Akt signaling pathway is controlled by NF-κB and has important functions in metabolic balance [91,92]. In another study involving streptozotocin-induced diabetic mice, ergosterol treatment attenuated mesangial cell proliferation and increases the matrix metalloproteinase-2 and -9 [90]. Furthermore, ergosterol alleviates ECM deposition by increasing transforming growth factor-β1 expression and Smad2 phosphorylation [93]. Ergosterol also increases glucose uptake by upregulating the expression and translocation activity of glucose transporter 4 and the phosphorylation of Akt and PKC in L6 muscle myoblast cells and different mouse tissues [89].

## 9. Neuroprotective Effects

Neuronal degeneration is a brain pathology characterized by protein aggregation and decline in the neuronal population, finally leading to neurodegenerative diseases such as Parkinson’s disease, Alzheimer’s disease, and amyotrophic lateral sclerosis [94]. Several risk factors are involved in neurodegeneration including oxidative stress, glial cell activation, neurotransmitter imbalance, and neuronal death [95]. Accumulating data demonstrate that ergosterol potentially serves as a neuroprotective agent by attenuating neuroinflammation and neuronal death. Ergosterol, at a concentration of 10 μg/mL (cal. 25.21 μM), showed an anti-neuroinflammatory effect on LPS-induced BV2 microglial cell activation by reducing the production of NO, a pro-inflammatory agent [57]. Moreover, ergosterol (50 nM), isolated from *Auricularia polytricha* edible mushroom, could suppress bisphenol-A-induced BV2 microglial cell inflammation by down-regulating the NF-κB signaling and enhancing the antioxidant pathway [96]. Furthermore, 100 nM of ergosterol exhibited a neuroprotective property against TNF-α-induced HT-22 hippocampal cell damage by promoting Nrf2/SOD-1 and RICTOR/Akt/GSK-3β signaling pathways and inhibiting ionotropic glutamate receptor overexpression via EGR-1 regulation [97]. Therefore, ergosterol seems to provide therapeutic effects that benefit neurodegenerative patients.

## 10. Antimicrobial Activity

The increased incidence of new microbial infections and the evolution of antibiotic-resistant strains have led scientists to develop new bioactive compounds from natural resources as alternatives for treating infectious diseases [98]. Ergosterol and ergosterol peroxide exhibit a broad range of antimicrobial activities [99]. For instance, the growth of *Helicobacter pylori* is inhibited by ergosterol; the minimum inhibitory concentrations are 10–20 μg/mL [100], suggesting that ergosterol has the potential to serve as an effective drug for gastritis. Ergosterol also exhibits antifungal activity against *Aspergillus flavus*, *Pencillium digitatum*, and *Fusarium vericilloides* [99]. *Trypanosoma cruzi* infection is one of the causes of the high incidence of Chagas disease, which is a life-threatening disease. Ergosterol causes rapid plasma membrane and mitochondrial permeabilization owing to alterations in membrane composition, resulting in the disruption and death of trypomastigotes [101]. Other studies have also revealed that the antibacterial effect of ergosterol is mediated by the disruption of the electron transport chain and oxidative phosphorylation via the disorganization of membrane fluidity, leading to enhanced membrane permeability [102,103,104]. These mechanisms may be advantageous and useful in the treatment of infectious diseases. In a recent study on the anti-HIV-1-protease activity of the hexane extract of the edible mushroom *Auricularia polytricha*, a phytochemical investigation revealed the presence of ergosterol, apart from other components such as linoleic acid and two other lipopolysaccharides, as the principal component in the extract [19]. Additionally, an in silico study suggested that ergosterol may be a potential candidate for treatment of COVID-19 because of its potential to inhibit SAR-CoV-2 main protease [41]. Nevertheless, further study is required to prove this hypothesis.

## 11. Anti-Hepatic Steatosis Effect

In addition to the beneficial effects mentioned above, ergosterol may have therapeutic potential for hepatic steatosis, which is the first step in the development of nonalcoholic fatty liver disease, steatohepatitis, fibrosis, cirrhosis, and hepatic carcinoma. Ergosterol reduces intracellular triglyceride accumulation in HepG2 cells treated with fatty acid. Its mechanism of action involves the direct induction of AMPK and acetyl-CoA carboxylase phosphorylation [35], which regulates lipid metabolism and cellular lipid homeostasis [105].

## 12. Future Perspective and Conclusions

Ergosterol is a versatile compound found in natural sources, especially in edible mushrooms. Continuous pharmacological research on ergosterol may lead to the development of new drugs and nutraceuticals for therapeutic use and promoting health. For example, ergosterol can be formulated as a novel antioxidant or anti-inflammatory agent. It may also be used in combination with other drugs, especially chemotherapeutic agents, to enhance drug efficacy and reduce side effects. Ergosterol-containing nutraceuticals can promote immune function, liver health, and overall well-being. Further studies are required to fully explore its mechanisms of action, and to understand its effects on the human body. In particular, animal studies and clinical trials must be encouraged. Formulation optimization should also be considered in order to increase ergosterol bioavailability.

## Figures and Tables

**Table 1:** Source of ergosterol from edible mushrooms.

Family	Species	Common Name	Reference
Agaricaceae	*Agaricus bisporus*	White button mushroom	[17]
	*Agaricus blazei*	Sun mushroom	[18]
Auriculariaceae	*Auricularia auricula-judae*	Wood ear mushroom	[17]
	*Auricularia polytricha*	Wood ear mushroom	[19,20]
Lycoperdacea	*Calvatia excipuliformis*	Pestle puffball	[21]
Agaricaceae	*Coprinus comatus*	Shaggy inkcap	[17]
Ganodermataceae	*Amauroderma rugosum*	Blood linzhi	[22]
	*Ganoderma lucidum*	Lingzhi, Reishi	[17,23]
Meripilaceae	*Grifola frondosa*	Maitake	[24,25]
Hericiaceae	*Hericium erinaceus*	Bearded tooth mushroom	[17]
	*Hericium novae-zealandiae*	Pekepekekiore	[26]
Hymenochaetaceae	*Inonotus obliquus*	Chaga mushroom	[27]
Russulaceae	*Lactarius deliciosus*	Saffron milk cap	[28]
	*Lactarius sanguifluus*	Bloody milk cap	[28]
	*Lactarius semisanguifluus*	Semi-bloody milk cap	[28]
	*Russula delica*	Milk-white brittlegill mushroom	[28]
Hydnangiaceae	*Laccaria amethystina*	Amethyst deceiver mushroom	[21]
	*Laccaria laccata*	Deceiver mushroom	[21]
Polyporaceae	*Laetiporus sulphureus*	Chicken of the woods	[21]
Boletaceae	*Leccinum scabrum*	Brown birch bolete	[21]
Agaricaceae	*Lycoperdon perlatum*	Gem-studded puffball	[21]
Marasmiaceae	*Lentinula edodes*	Shiitake	[17,29,30]
Agaricaceae	*Macrolepiota procera*	Parasol mushroom	[21]
Marasmiaceae	*Marasmius oreades*	Fairy ring champignon	[21]
Polyporaceae	*Neolentinus lepideus*	Scaly sawgill	[31]
Pleurotaceae	*Pleurotus citrinopileatus*	Golden oyster mushroom	[32]
	*Pleurotus eryngii*	King oyster mushroom	[17]
	*Pleurotus ostreatus*	Oyster mushroom	[17]
	*Pleurotus pulmonarius*	Indian Oyster mushroom	[33,34]
Polyporaceae	*Poria cocos* Wolf	Fu-ling, Indian bread	[35]
Rhizopogonaceae	*Rhizopogon luteolus*	Yellow false truffle	[36]
Sparassidaceae	*Sparassis crispa*	Cauliflower fungus	[37]
Suillaceae	*Suillus bellinii*	Champagne bolete	[28]
	*Suillus variegatus*	Velvet bolete	[21]
Boletaceae	*Xerocomus badius*	Bay bolete	[21]

**Table 2:** Outcome and mechanism of antioxidant effect of ergosterol.

Subject/Model	Dose	Outcome and Mechanism	Reference
tert-Butyl hydroperoxide-induced *Saccharomyces cerevisiae*	0.83 mM	Lipid peroxidation ^$^	[44]
In Vitro non-cell-based assay	11 μM	DPPH radical-scavenging activity ^#^	[44]
Computational analysis (Gaussian 16 program)	N/A	Electron transfer followed by proton transfer mechanism ^#^	[44]
In Vitro non-cell-based assays	2 μg/mL	Lipid peroxidation ^$^	[45]
H_2_O_2_-induced primary dermal fibroblast (PCS-201-012)	200 and 400 μg/mL	Intracellular ROS accumulation ^$^	[45]
LPS-induced Sprague Dawley rats	25 and 50 mg/kg	Nrf2/HO-1 signaling ^#^ SOD level and activity ^#^ MDA level ^$^	[46]
LPS-treated H9C2 myoblast cells	5–20 μM	Nrf2/HO-1 signaling ^#^ SOD activity ^#^ MDA level ^$^	[46]

Abbreviations: DPPH, 2,2-diphenyl-1-picrylhydrazyl; N/A, not applicable; Nrf2, nuclear factor erythroid 2-related factor 2; HO-1, heme oxygenase-1; LPS, lipopolysaccharide; ROS, reactive oxygen species; SOD, superoxide dismutase; MDA, malondialdehyde; ^#^, increase; ^$^, decrease.

## Data Availability

The data used to support the findings of this study can be made available by the corresponding author upon request.

## References

[1] Bell V., Silva C., Guina J., Fernandes T.H. (2022). Mushrooms as future generation healthy foods. Front. Nutr..

[2] Kumar K., Mehra R., Guiné R.P.F., Lima M.J., Kumar N., Kaushik R., Ahmed N., Yadav A.N., Kumar H. (2021). Edible Mushrooms: A Comprehensive Review on Bioactive Compounds with Health Benefits and Processing Aspects. Foods.

[3] Baker M.A.B., Brown A.J. (2019). A detour to sterol synthesis. Nat. Microbiol..

[4] Yaoita Y., Matsuki K., Iijima T., Nakano S., Kakuda R., Machida K., Kikuchi M. (2001). New sterols and triterpenoids from four edible mushrooms. Chem. Pharm. Bull..

[5] Haubrich B.A. (2018). Microbial Sterolomics as a Chemical Biology Tool. Molecules.

[6] Villares A., García-Lafuente A., Guillamón E., Ramos Á. (2012). Identification and quantification of ergosterol and phenolic compounds occurring in Tuber spp. truffles. J. Food Compos. Anal..

[7] Kennel K.A., Drake M.T., Hurley D.L. (2010). Vitamin D deficiency in adults: When to test and how to treat. Mayo Clin. Proc..

[8] Quackenbush F.W., Peterson W.H., Steenbock H. (1935). A Study of the Nutritive Value of Mushrooms: Five Figures. J. Nutr..

[9] Shao S., Hernandez M., Kramer J.K., Rinker D.L., Tsao R. (2010). Ergosterol profiles, fatty acid composition, and antioxidant activities of button mushrooms as affected by tissue part and developmental stage. J. Agric. Food Chem..

[10] Cardwell G., Bornman J.F., James A.P., Black L.J. (2018). A Review of Mushrooms as a Potential Source of Dietary Vitamin D. Nutrients.

[11] Barreira J.C.M., Oliveira M.B.P.P., Ferreira I.C.F.R. (2014). Development of a Novel Methodology for the Analysis of Ergosterol in Mushrooms. Food Anal. Methods.

[12] Teichmann A., Dutta P.C., Staffas A., Jägerstad M. (2007). Sterol and vitamin D2 concentrations in cultivated and wild grown mushrooms: Effects of UV irradiation. LWT—Food Sci. Technol..

[13] Feng S., Wang L., Belwal T., Li L., Luo Z. (2020). Phytosterols extraction from hickory (Carya cathayensis Sarg.) husk with a green direct citric acid hydrolysis extraction method. Food Chem..

[14] Ghisoni S., Lucini L., Rocchetti G., Chiodelli G., Farinelli D., Tombesi S., Trevisan M. (2020). Untargeted metabolomics with multivariate analysis to discriminate hazelnut (*Corylus avellana* L.) cultivars and their geographical origin. J. Sci. Food Agric..

[15] Ahmed S.B., Hamed M.S., Khiralla G.M., Mohamed A.F. (2020). Cactus and lupin extracts as prospective anticancer agents compared with utoral drug. J. Food Biochem..

[16] Keser S. (2014). Antiradical activities and phytochemical compounds of firethorn (*Pyracantha coccinea*) fruit extracts. Nat. Prod. Res..

[17] Poniedziałek B., Siwulski M., Wiater A., Komaniecka I., Komosa A., Gąsecka M., Magdziak Z., Mleczek M., Niedzielski P., Proch J. (2019). The Effect of Mushroom Extracts on Human Platelet and Blood Coagulation: In vitro Screening of Eight Edible Species. Nutrients.

[18] Corrêa R.C.G., Barros L., Fernandes Â., Sokovic M., Bracht A., Peralta R.M., Ferreira I. (2018). A natural food ingredient based on ergosterol: Optimization of the extraction from Agaricus blazei, evaluation of bioactive properties and incorporation in yogurts. Food Funct..

[19] Sillapachaiyaporn C., Nilkhet S., Ung A.T., Chuchawankul S. (2019). Anti-HIV-1 protease activity of the crude extracts and isolated compounds from Auricularia polytricha. BMC Complement. Altern. Med..

[20] Sillapachaiyaporn C., Rangsinth P., Nilkhet S., Ung A.T., Chuchawankul S., Tencomnao T. (2021). Neuroprotective Effects against Glutamate-Induced HT-22 Hippocampal Cell Damage and Caenorhabditis elegans Lifespan/Healthspan Enhancing Activity of Auricularia polytricha Mushroom Extracts. Pharmaceuticals.

[21] Nowak R., Nowacka-Jechalke N., Pietrzak W., Gawlik-Dziki U. (2022). A new look at edible and medicinal mushrooms as a source of ergosterol and ergosterol peroxide—UHPLC-MS/MS analysis. Food Chem..

[22] Li J., Cheng Y., Li R., Wu X., Zheng C., Shiu P.H.-T., Chan J.C.-K., Rangsinth P., Liu C., Leung S.W.-S. (2022). Protective Effects of Amauroderma rugosum on Doxorubicin-Induced Cardiotoxicity through Suppressing Oxidative Stress, Mitochondrial Dysfunction, Apoptosis, and Activating Akt/mTOR and Nrf2/HO-1 Signaling Pathways. Oxidative Med. Cell. Longev..

[23] Martínez-Montemayor M.M., Ling T., Suárez-Arroyo I.J., Ortiz-Soto G., Santiago-Negrón C.L., Lacourt-Ventura M.Y., Valentín-Acevedo A., Lang W.H., Rivas F. (2019). Identification of Biologically Active Ganoderma lucidum Compounds and Synthesis of Improved Derivatives That Confer Anti-cancer Activities in vitro. Front. Pharm..

[24] Kawai J., Mori K., Hirasawa N. (2019). Grifola frondosa extract and ergosterol reduce allergic reactions in an allergy mouse model by suppressing the degranulation of mast cells. Biosci. Biotechnol. Biochem..

[25] Kawai J., Higuchi Y., Hirota M., Hirasawa N., Mori K. (2018). Ergosterol and its derivatives from Grifola frondosa inhibit antigen-induced degranulation of RBL-2H3 cells by suppressing the aggregation of high affinity IgE receptors. Biosci. Biotechnol. Biochem..

[26] Chen Z.G., Bishop K.S., Tanambell H., Buchanan P., Smith C., Quek S.Y. (2019). Characterization of the bioactivities of an ethanol extract and some of its constituents from the New Zealand native mushroom Hericium novae-zealandiae. Food Funct..

[27] Ma L., Chen H., Dong P., Lu X. (2013). Anti-inflammatory and anticancer activities of extracts and compounds from the mushroom Inonotus obliquus. Food Chem..

[28] Kalogeropoulos N., Yanni A.E., Koutrotsios G., Aloupi M. (2013). Bioactive microconstituents and antioxidant properties of wild edible mushrooms from the island of Lesvos, Greece. Food Chem. Toxicol. Int. J. Publ. Br. Ind. Biol. Res. Assoc..

[29] Morales D., Tejedor-Calvo E., Jurado-Chivato N., Polo G., Tabernero M.D., Ruíz-Rodríguez A., Largo C., Soler-Rivas C.J.F. (2019). In vitro and in vivo testing of the hypocholesterolemic activity of ergosterol- and β-glucan-enriched extracts obtained from shiitake mushrooms (*Lentinula edodes*). Food Funct..

[30] Drori A., Shabat Y., Ben Ya’acov A., Danay O., Levanon D., Zolotarov L., Ilan Y. (2016). Extracts from Lentinula edodes (Shiitake) Edible Mushrooms Enriched with Vitamin D Exert an Anti-Inflammatory Hepatoprotective Effect. J. Med. Food.

[31] Quintero-Cabello K.P., Palafox-Rivera P., Lugo-Flores M.A., Gaitán-Hernández R., González-Aguilar G.A., Silva-Espinoza B.A., Tortoledo-Ortiz O., Ayala-Zavala J.F., Monribot-Villanueva J.L., Guerrero-Analco J.A. (2021). Contribution of Bioactive Compounds to the Antioxidant Capacity of the Edible Mushroom Neolentinus lepideus. Chem. Biodivers..

[32] Hu S.H., Liang Z.C., Chia Y.C., Lien J.L., Chen K.S., Lee M.Y., Wang J.C. (2006). Antihyperlipidemic and antioxidant effects of extracts from Pleurotus citrinopileatus. J. Agric. Food Chem..

[33] Milovanovic I., Zengin G., Maksimovic S., Tadic V. (2021). Supercritical and ultrasound-assisted extracts from Pleurotus pulmonarius mushroom: Chemical profiles, antioxidative, and enzyme-inhibitory properties. J. Sci. Food Agric..

[34] Abidin M.H., Abdullah N., Abidin N.Z. (2016). Protective Effect of Antioxidant Extracts from Grey Oyster Mushroom, Pleurotus pulmonarius (Agaricomycetes), Against Human Low-Density Lipoprotein Oxidation and Aortic Endothelial Cell Damage. Int. J. Med. Mushrooms.

[35] Kim J.H., Sim H.A., Jung D.Y., Lim E.Y., Kim Y.T., Kim B.J., Jung M.H. (2019). Poria cocus Wolf Extract Ameliorates Hepatic Steatosis through Regulation of Lipid Metabolism, Inhibition of ER Stress, and Activation of Autophagy via AMPK Activation. Int. J. Mol. Sci..

[36] Tel-Çayan G., Muhammad A., Duru M.E., Öztürk M., Adhikari A., Türkoğlu A. (2016). A new fatty acid ester from an edible mushroom Rhizopogon luteolus. Nat. Prod. Res..

[37] Sułkowska-Ziaja K., Muszyńska B., Szewczyk A. (2015). Antioxidant components of selected indigenous edible mushrooms of the obsolete order Aphyllophorales. Rev. Iberoam. Micol..

[38] Zhao Y.Y., Cheng X.L., Liu R., Ho C.C., Wei F., Yan S.H., Lin R.C., Zhang Y., Sun W.J. (2011). Pharmacokinetics of ergosterol in rats using rapid resolution liquid chromatography-atmospheric pressure chemical ionization multi-stage tandem mass spectrometry and rapid resolution liquid chromatography/tandem mass spectrometry. J. Chromatogr. B Anal. Technol. Biomed. Life Sci..

[39] Dong Z., Iqbal S., Zhao Z. (2020). Preparation of ergosterol-loaded nanostructured lipid carriers for enhancing oral bioavailability and antidiabetic nephropathy effects. AAPS PharmSciTech.

[40] Zhang H.Y., Firempong C.K., Wang Y.W., Xu W.Q., Wang M.M., Cao X., Zhu Y., Tong S.S., Yu J.N., Xu X.M. (2016). Ergosterol-loaded poly(lactide-co-glycolide) nanoparticles with enhanced in vitro antitumor activity and oral bioavailability. Acta Pharmacol. Sin..

[41] Rangsinth P., Sillapachaiyaporn C., Nilkhet S., Tencomnao T., Ung A.T., Chuchawankul S. (2021). Mushroom-derived bioactive compounds potentially serve as the inhibitors of SARS-CoV-2 main protease: An in silico approach. J. Tradit. Complement. Med..

[42] Pizzino G., Irrera N., Cucinotta M., Pallio G., Mannino F., Arcoraci V., Squadrito F., Altavilla D., Bitto A. (2017). Oxidative Stress: Harms and Benefits for Human Health. Oxid. Med. Cell Longev..

[43] Ray P.D., Huang B.W., Tsuji Y. (2012). Reactive oxygen species (ROS) homeostasis and redox regulation in cellular signaling. Cell. Signal..

[44] Dupont S., Fleurat-Lessard P., Cruz R.G., Lafarge C., Grangeteau C., Yahou F., Gerbeau-Pissot P., Abrahão Júnior O., Gervais P., Simon-Plas F. (2021). Antioxidant Properties of Ergosterol and Its Role in Yeast Resistance to Oxidation. Antioxidants.

[45] Yongxia Z., Jian X., Suyuan H., Aixin N., Lihong Z. (2020). Isolation and characterization of ergosterol from Monascus anka for anti-lipid peroxidation properties. J. Mycol. Médicale.

[46] Xu J., Lin C., Wang T., Zhang P., Liu Z., Lu C. (2018). Ergosterol attenuates LPS-induced myocardial injury by modulating oxidative stress and apoptosis in rats. Cell. Physiol. Biochem..

[47] Moro C., Palacios I., Lozano M., D’Arrigo M., Guillamón E., Villares A., Martínez J.A., García-Lafuente A. (2012). Anti-inflammatory activity of methanolic extracts from edible mushrooms in LPS activated RAW 264.7 macrophages. Food Chem..

[48] Huan W., Tianzhu Z., Yu L., Shumin W. (2017). Effects of Ergosterol on COPD in Mice via JAK3/STAT3/NF-κB Pathway. Inflammation.

[49] Sun X., Feng X., Zheng D., Li A., Li C., Li S., Zhao Z. (2019). Ergosterol attenuates cigarette smoke extract-induced COPD by modulating inflammation, oxidative stress and apoptosis in vitro and in vivo. Clin. Sci..

[50] Kobori M., Yoshida M., Ohnishi-Kameyama M., Shinmoto H. (2007). Ergosterol peroxide from an edible mushroom suppresses inflammatory responses in RAW264. 7 macrophages and growth of HT29 colon adenocarcinoma cells. Br. J. Pharmacol..

[51] Yoo M.-S., Shin J.-S., Choi H.-E., Cho Y.-W., Bang M.-H., Baek N.-I., Lee K.-T. (2012). Fucosterol isolated from Undaria pinnatifida inhibits lipopolysaccharide-induced production of nitric oxide and pro-inflammatory cytokines via the inactivation of nuclear factor-κB and p38 mitogen-activated protein kinase in RAW264. 7 macrophages. Food Chem..

[52] Yuan L., Zhang F., Shen M., Jia S., Xie J. (2019). Phytosterols suppress phagocytosis and inhibit inflammatory mediators via ERK pathway on LPS-triggered inflammatory responses in RAW264. 7 macrophages and the correlation with their structure. Foods.

[53] Xu J., Xiao C., Xu H., Yang S., Chen Z., Wang H., Zheng B., Mao B., Wu X. (2021). Anti-inflammatory effects of Ganoderma lucidum sterols via attenuation of the p38 MAPK and NF-κB pathways in LPS-induced RAW 264.7 macrophages. Food Chem. Toxicol..

[54] Al-Rabia M.W., Mohamed G.A., Ibrahim S.R.M., Asfour H.Z. (2021). Anti-inflammatory ergosterol derivatives from the endophytic fungus Fusarium chlamydosporum. Nat. Prod. Res..

[55] Zheng M.S., Hwang N.K., Kim D.H., Moon T.C., Son J.K., Chang H.W. (2008). Chemical constituents of Melandrium firmum Rohrbach and their anti-inflammatory activity. Arch. Pharmacal Res..

[56] Hong Y.-J., Jang A., Jang H.-J., Yang K.-S. (2012). Inhibition of nitric oxide production, iNOS and COX-2 expression of ergosterol derivatives from Phellinus pini. Nat. Prod. Sci..

[57] Nallathamby N., Guan-Serm L., Vidyadaran S., Malek S.N.A., Raman J., Sabaratnam V. (2015). Ergosterol of Cordyceps militaris attenuates LPS induced inflammation in BV2 microglia cells. Nat. Prod. Commun..

[58] Zhang S.-Y., Xu L.-T., Li A.-X., Wang S.-M. (2015). Effects of ergosterol, isolated from Scleroderma polyrhizum Pers., on lipopolysaccharide-induced inflammatory responses in acute lung injury. Inflammation.

[59] Ikarashi N., Hoshino M., Ono T., Toda T., Yazawa Y., Sugiyama K. (2020). A mechanism by which ergosterol inhibits the promotion of bladder carcinogenesis in rats. Biomedicines.

[60] Park H., Lee T.H., Chang F., Kwon H.J., Kim J., Kim H. (2013). Synthesis of ergosterol and 5, 6-dihydroergosterol glycosides and their inhibitory activities on lipopolysaccharide-induced nitric oxide production. Bull. Korean Chem. Soc..

[61] Kuo C.-F., Hsieh C.-H., Lin W.-Y. (2011). Proteomic response of LAP-activated RAW 264.7 macrophages to the anti-inflammatory property of fungal ergosterol. Food Chem..

[62] Ano Y., Kutsukake T., Hoshi A., Yoshida A., Nakayama H. (2015). Identification of a novel dehydroergosterol enhancing microglial anti-inflammatory activity in a dairy product fermented with Penicillium candidum. PLoS ONE.

[63] Liu C., Zhao S., Zhu C., Gao Q., Bai J., Si J., Chen Y. (2020). Ergosterol ameliorates renal inflammatory responses in mice model of diabetic nephropathy. Biomed. Pharmacother..

[64] Kushairi N., Tarmizi N.A.K.A., Phan C.W., Macreadie I., Sabaratnam V., Naidu M., David P. (2020). Modulation of neuroinflammatory pathways by medicinal mushrooms, with particular relevance to Alzheimer’s disease. Trends Food Sci. Technol..

[65] Erol E., Ali Z., Oztürk M., Khan S., Khan I.A. (2020). Inhibition of iNOS induction and nf-κΒ activation by taste compounds from the edible mushroom *Tricholoma caligatum* (Viv.) ricken. Rec. Nat. Prod..

[66] Wu S.-J., Lu T.-M., Lai M.-N., Ng L.-T. (2013). Immunomodulatory activities of medicinal mushroom Grifola frondosa extract and its bioactive constituent. Am. J. Chin. Med..

[67] Kim S.J., Shin H.J., Lee G.H., Kim D.S., Kim H.L., Park J., Jung Y., Youn D.H., Kang J., Hong S.H. (2015). Beneficial effects of the traditional medicine Igongsan and its constituent ergosterol on dextran sulfate sodium-induced colitis in mice. Mol. Med. Rep..

[68] Durmus A., Durmus I., Bender O., Karatepe O. (2021). The effect of Hericium erinaceum on the prevention of chemically induced experimental colitis in rats. Korean J. Intern Med..

[69] Kageyama-Yahara N., Wang P., Wang X., Yamamoto T., Kadowaki M. (2010). The inhibitory effect of ergosterol, a bioactive constituent of a traditional Japanese herbal medicine saireito on the activity of mucosal-type mast cells. Biol. Pharm. Bull..

[70] Kawai J., Andoh T., Mori K. (2021). Suppression of leukotriene B4 production is involved in the anti-pruritic action of Grifola frondosa in pollen allergy-induced ocular itching in mice. Food Agric. Immunol..

[71] Huang M., Lu J.J., Ding J. (2021). Natural Products in Cancer Therapy: Past, Present and Future. Nat. Prod. Bioprospecting.

[72] Choudhari A.S., Mandave P.C., Deshpande M., Ranjekar P., Prakash O. (2019). Phytochemicals in Cancer Treatment: From Preclinical Studies to Clinical Practice. Front. Pharmacol..

[73] Ashraf M.A. (2020). Phytochemicals as Potential Anticancer Drugs: Time to Ponder Nature’s Bounty. BioMed Res. Int..

[74] Chen S., Yong T., Zhang Y., Su J., Jiao C., Xie Y. (2017). Anti-tumor and Anti-angiogenic Ergosterols from Ganoderma lucidum. Front. Chem..

[75] Wu H.Y., Yang F.L., Li L.H., Rao Y.K., Ju T.C., Wong W.T., Hsieh C.Y., Pivkin M.V., Hua K.F., Wu S.H. (2018). Ergosterol peroxide from marine fungus Phoma sp. induces ROS-dependent apoptosis and autophagy in human lung adenocarcinoma cells. Sci. Rep..

[76] Sana T., Siddiqui B.S., Shahzad S., Farooq A.D., Siddiqui F., Sattar S., Begum S. (2019). Antiproliferative Activity and Characterization of Metabolites of *Aspergillus nidulans*: An Endophytic Fungus from Nyctanthes arbor-tristis Linn. Against Three Human Cancer Cell Lines. Med. Chem..

[77] Subbiah M.T., Abplanalp W. (2003). Ergosterol (major sterol of baker’s and brewer’s yeast extracts) inhibits the growth of human breast cancer cells in vitro and the potential role of its oxidation products. Int. J. Vitam. Nutr. Res..

[78] Hao J., Zhang X., Yu W., Wang R., Xue Z., Kou X. (2017). Identification and Evaluation of Bioactivity of Compounds from the Mushroom *Pleurotus nebrodensis* (Agaricomycetes) against Breast Cancer. Int. J. Med. Mushrooms.

[79] Pluchino L.A., Liu A.K., Wang H.C. (2015). Reactive oxygen species-mediated breast cell carcinogenesis enhanced by multiple carcinogens and intervened by dietary ergosterol and mimosine. Free Radic. Biol. Med..

[80] Chen L.Y., Sheu M.T., Liu D.Z., Liao C.K., Ho H.O., Kao W.Y., Ho Y.S., Lee W.S., Su C.H. (2011). Pretreatment with an ethanolic extract of *Taiwanofungus camphoratus* (*Antrodia camphorata*) enhances the cytotoxic effects of amphotericin B. J. Agric. Food Chem..

[81] Lin Y.C., Lee B.H., Alagie J., Su C.H. (2017). Combination treatment of ergosterol followed by amphotericin B induces necrotic cell death in human hepatocellular carcinoma cells. Oncotarget.

[82] Li X., Wu Q., Xie Y., Ding Y., Du W.W., Sdiri M., Yang B.B. (2015). Ergosterol purified from medicinal mushroom Amauroderma rude inhibits cancer growth in vitro and in vivo by up-regulating multiple tumor suppressors. Oncotarget.

[83] Yazawa Y., Ikarashi N., Hoshino M., Kikkawa H., Sakuma F., Sugiyama K. (2020). Inhibitory effect of ergosterol on bladder carcinogenesis is due to androgen signaling inhibition by brassicasterol, a metabolite of ergosterol. J. Nat. Med..

[84] Muñoz-Fonseca M.B., Vidal-Limon A., Fernández-Pomares C., Rojas-Durán F., Hernández-Aguilar M.E., Espinoza C., Trigos A., Suárez-Medellín J. (2021). Ergosterol exerts a differential effect on AR-dependent LNCaP and AR-independent DU-145 cancer cells. Nat. Prod. Res..

[85] Takaku T., Kimura Y., Okuda H. (2001). Isolation of an antitumor compound from Agaricus blazei Murill and its mechanism of action. J. Nutr..

[86] Tan W., Pan M., Liu H., Tian H., Ye Q., Liu H. (2017). Ergosterol peroxide inhibits ovarian cancer cell growth through multiple pathways. OncoTargets Ther..

[87] Hu J., Ye M., Zhou Z. (2017). Aptamers: Novel diagnostic and therapeutic tools for diabetes mellitus and metabolic diseases. J. Mol. Med..

[88] Azushima K., Gurley S.B., Coffman T.M. (2018). Modelling diabetic nephropathy in mice. Nat. Rev. Nephrol..

[89] Xiong M., Huang Y., Liu Y., Huang M., Song G., Ming Q., Ma X., Yang J., Deng S., Wen Y. (2018). Antidiabetic activity of ergosterol from Pleurotus ostreatus in KK-A(y) mice with spontaneous type 2 diabetes mellitus. Mol. Nutr. Food Res..

[90] Ang L., Yuguang L., Liying W., Shuying Z., Liting X., Shumin W. (2015). Ergosterol alleviates kidney injury in streptozotocin-induced diabetic mice. Evid.-Based Complement. Altern. Med..

[91] Holman G., Kasuga M. (1997). From receptor to transporter: Insulin signalling to glucose transport. Diabetologia.

[92] Vareda P.M.P., Saldanha L.L., Camaforte N.A.d.P., Violato N.M., Dokkedal A.L., Bosqueiro J.R. (2014). Myrcia bella leaf extract presents hypoglycemic activity via PI3k/Akt insulin signaling pathway. Evid.-Based Complement. Altern. Med..

[93] Dong Z., Sun Y., Wei G., Li S., Zhao Z. (2019). Ergosterol ameliorates diabetic nephropathy by attenuating mesangial cell proliferation and extracellular matrix deposition via the TGF-β1/Smad2 signaling pathway. Nutrients.

[94] Barnham K.J., Masters C.L., Bush A.I. (2004). Neurodegenerative diseases and oxidative stress. Nat. Rev. Drug Discov..

[95] Pardillo-Díaz R., Pérez-García P., Castro C., Nunez-Abades P., Carrascal L. (2022). Oxidative Stress as a Potential Mechanism Underlying Membrane Hyperexcitability in Neurodegenerative Diseases. Antioxidants.

[96] Sillapachaiyaporn C., Chuchawankul S., Nilkhet S., Moungkote N., Sarachana T., Ung A.T., Baek S.J., Tencomnao T. (2022). Ergosterol isolated from cloud ear mushroom (*Auricularia polytricha*) attenuates bisphenol A-induced BV2 microglial cell inflammation. Food Res. Int..

[97] Sillapachaiyaporn C., Mongkolpobsin K., Chuchawankul S., Tencomnao T., Baek S.J. (2022). Neuroprotective effects of ergosterol against TNF-α-induced HT-22 hippocampal cell injury. Biomed. Pharmacother..

[98] Khameneh B., Iranshahy M., Soheili V., Fazly Bazzaz B.S. (2019). Review on plant antimicrobials: A mechanistic viewpoint. Antimicrob. Resist. Infect. Control.

[99] Mbambo B., Odhav B., Mohanlall V.J.J.M.P.R. (2012). Antifungal activity of stigmasterol, sitosterol and ergosterol from Bulbine natalensis Baker (Asphodelaceae). J. Med. Plants Res..

[100] Li Y., Song Y.C., Liu J.Y., Ma Y.M., Tan R.X. (2005). Anti-Helicobacter pylori substances from endophytic fungal cultures. World J. Microbiol. Biotechnol..

[101] Alexandre T.R., Lima M.L., Galuppo M.K., Mesquita J.T., do Nascimento M.A., dos Santos A.L., Sartorelli P., Pimenta D.C., Tempone A.G. (2017). Ergosterol isolated from the basidiomycete Pleurotus salmoneostramineus affects Trypanosoma cruzi plasma membrane and mitochondria. J. Venom. Anim. Toxins Incl. Trop. Dis..

[102] Andrade J.C., Morais-Braga M.F., Guedes G.M., Tintino S.R., Freitas M.A., Menezes I.R., Coutinho H.D. (2014). Enhancement of the antibiotic activity of aminoglycosides by alpha-tocopherol and other cholesterol derivates. Biomed. Pharmacother. Biomed. Pharmacother..

[103] Andrade J.C., Morais Braga M.F.B., Guedes G.M.M., Tintino S.R., Freitas M.A., Quintans L.J., Menezes I.R.A., Coutinho H.D.M. (2018). Cholecalciferol, Ergosterol, and Cholesterol Enhance the Antibiotic Activity of Drugs. Int. J. Vitam. Nutr. Res. Int. Z. Fur Vitam.—Ernahrungsforschung. J. Int. Vitaminol. Nutr..

[104] Tintino S.R., Oliveira-Tintino C.D., Campina F.F., Costa M.S., Cruz R.P., Pereira R.L., Andrade J.C., Sousa E.O., Siqueira-Junior J.P., Coutinho H.D. (2017). Cholesterol and ergosterol affect the activity of Staphylococcus aureus antibiotic efflux pumps. Microb. Pathog..

[105] Fediuc S., Gaidhu M.P., Ceddia R.B. (2006). Regulation of AMP-activated protein kinase and acetyl-CoA carboxylase phosphorylation by palmitate in skeletal muscle cells. J. Lipid Res..

